# Acute Non-Suppurative Encephalitis

**Published:** 1918-11

**Authors:** 


**Acute Non-Suppurative Encephalitis.** Creyx, Jour. de Med. de Bordeaux, Mch., reports a case fatal after 4 days, temperature ranging from 40 to 42 (104-107.6 F.) in which syphilis, typhoid, tuberculosis, diabetic and nephritic coma were excluded and blood cultures negative. No exact etiologic diagnosis made. According to etiology, he submits the following classification: Malarial, Syphilitic, Hydrophobic, Disease of Heine-Medin, Tuberculous, Streptococcic, Straphylococcic, Pneumococcic and infections with other bacteria, not specific. In case of association suppuration is the rule. As to symptoms and localization, he classifies thus: Diffuse—delirious, convulsive, comatose. Focal—partial epilepsy, localized, choreiform movements; Paralyses characterized by syndromes varying according to level of the neuraxis—cortical, meso-cephalic, Wernicke's acute superior polioencephalitis, bulbo-protuberantial, acute bulbar, cerebellar; participation of the spinal marrow—encephalo-myelitis. According to age: Foetal (Little's Disease?), Infantile, common syndromes of infantile hemiplegia or diplegia—Chorea of Sydenham, Milan, Grabois?, sequellar forms presenting a number of destructive and cicatricial localizations, Encephalic forms of certain sceroses in plaques.

